# Anthropogenic and Environmental Factors Determining Local Favourable Conditions for Wolves during the Cold Season

**DOI:** 10.3390/ani11071895

**Published:** 2021-06-25

**Authors:** Paolo Viola, Settimio Adriani, Carlo Maria Rossi, Cinzia Franceschini, Riccardo Primi, Marco Apollonio, Andrea Amici

**Affiliations:** 1 Department of Agricultural and Forest Sciences, University of Tuscia, Via S. C. de Lellis snc, 01100 Viterbo, VT, Italy; p.viola82@unitus.it (P.V.); adrianisettimio@libero.it (S.A.); carlomariarossivt@gmail.com (C.M.R.); cinziafranceschini@msn.com (C.F.); primi@unitus.it (R.P.); 2 Department of Biological, Geological and Environmental Science, University of Bologna, Piazza di Porta S. Donato 1, 40127 Bologna, BO, Italy; 3 Department of Veterinary Medicine, University of Sassari, 07100 Sassari, SS, Italy; marcoapo@uniss.it

**Keywords:** anthropogenic opportunities, wolf–free-ranging-dogs interaction, heat load index, human disturbance, resource availability, thermal refuges, wolf howling, audibility analysis, LASSO regression

## Abstract

**Simple Summary:**

Wolves normally howl in response to unfamiliar vocalisations, to defend their territory and the important resources within it (e.g., pups and prey). During the non-rendezvous period (late autumn and winter), the protectiveness of adults towards pups decreases, as well as reactions to unfamiliar vocal stimuli. In the late fall of 2010, we performed a saturation wolf howling design in the Cicolano area (Central Apennines, Italy), aiming to identify environmental and human-related characteristics of locations where wolves are prone to respond to unfamiliar howling and to assess their eventual ability to provide insights into the distribution of valuable resources (aside from pups) during the cold season. We found that winter response sites (WRS) were characterized by diverging conditions, with respect to all available sites, suggesting that they are non-randomly located but, instead, had been selected by wolves for some reason. We recorded a positive role of thermal refuges and the occurrence of wild boar drive hunts, as well as the negative roles of other forms of human presence and activities, including the occurrence of free-ranging dogs. These results could be of interest both for conservation purposes and for assessing interactions with human activities.

**Abstract:**

Winter resources are crucial for wildlife, and, at a local scale, some anthropogenic and environmental factors could affect their availability. In the case of wolves, it is known that vocalisations in response to unfamiliar howls are issued to defend their territory and the important resources within it. Then, we studied the characteristics of winter response sites (WRS) during the cold season, aiming to assess their eventual ability to provide insights into the distribution of valuable resources within their territories. Within this scope, we planned a wolf-howling survey following a standardised approach. The study covered an Apennine (Central Italy) area of 500 km^2^. A hexagonal mesh was imposed on the area, in order to determine the values of different variables at the local scale. A logistic LASSO regression was performed. WRS were positively related to the presence of thermal refuges (odds = 114.485), to patch richness (odds = 1.153), wild boar drive hunting areas (odds = 1.015), and time elapsed since the last hunt (odds = 1.019). Among negative factors, stray dogs reply considerably affects wolves’ responsiveness (odds = 0.207), where odds are the exponentiated coefficients estimated by the logistic lasso regression. These results suggest that WRS are related to anthropogenic and environmental factors favouring the predation process.

## 1. Introduction

Wolves are prone to react to vocal stimuli for territorial maintenance and the defence of resources within it, aiming to minimize the risk of direct aggressive interactions between packs [1,2]; however, wolf howling elicitation is commonly described as infrequent and sporadic [1,3,4]. In general, as suggested by these authors, wolves are reluctant to reveal their presence to potential invaders, to escape the risk of deliberate attacks, and decide to respond where precious resources or niches of their territory justify the activation of protective behaviours.

Among such important resources, pups and kills are the most motivating [1,5]. Accordingly, the response rate (RR) shows a temporal pattern related to the yearly biological cycle. The RR has been reported as: (i) low when pups are in the den; (ii) high when pups are raised at rendezvous sites; and (iii) decreasing in late fall and winter, with a minimum reached in December, when the young wolves are grown up and follow adults [3,4].

Then, during the non-rendezvous period (in particular, December–February), adult protectiveness towards pups decreases and wolf reactions to unfamiliar vocal stimuli become prevalent at kill and scavenging sites [1,2]. In this period, non-significant differences in response rates between packs and lone wolves were also reported [3].

While several studies have used both spontaneous and elicited wolf howling locations, as well as other presence indices, to assess the general habitat suitability at the home range scale [6,7,8,9,10], response locations have rarely been used, during the summer period, as a possible indicator of dens and rendezvous site locations [11,12,13]; however, no attention has been paid to late fall or winter response sites (WRS). According to the above considerations, in the present study, we focus our attention on WRS characteristics, aiming to assess their eventual ability to provide insights into the distribution of valuable resources (aside from dens and pups) within their territories. As, among all the factors potentially affecting wolf presence, prey and carrion are motivating resources (especially during late fall and winter [1]), we devoted particular attention to environmental, landscape, and anthropogenic factors related to prey availability and hunting efficiency in WRS. Indeed, McPhee et al. [14] suggested that predation is a “hierarchical process,” where predators choose their hunting area primarily on the basis of prey abundance and predictability, and secondarily on the basis of environmental factors and landscape features favouring prey detectability and attack success. Some authors have even suggested that kill site occurrence is more affected by habitat attributes than prey abundance [15,16,17].

In particular, the aims of this study are to: (i) analyse the characteristics of WRS; (ii) verify the occurrence of diverging conditions in WRS with an approach presence (reply) vs. availability; and (iii) identify landscape attributes, human-related and environmental factors positively or negatively affecting WRS occurrence.

Advancement in knowledge in this topic could be important in a bivalent way: (i) for planning, projecting, and managing suitable protection areas from a top predator conservation perspective; and (ii) for predicting the wintering distribution of the species and spatializing the risk of interaction with human activity in a conflict management perspective. Furthermore, it is highly important to clarify the role of some human activities, such as drive hunting, anecdotally considered a source of disturbance.

## 2. Materials and Methods

### 2.1. Study Area

**Figure 1:**
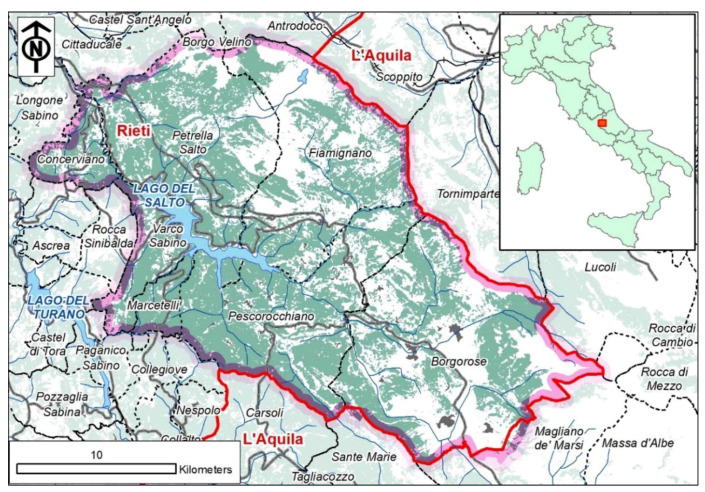
Map of the study area belonging to the “Salto Cicolano” mountain community (Rieti Province, Lazio Region). Regional border in red. Forest cover in green.

The study was conducted in the eastern part of the province of Rieti (Lazio Region, Central Italy), within an Apennine area of 500 km^2^ belonging to the “Salto Cicolano” mountain community which intersects seven municipalities (Petrella Salto, Fiamignano, Concerviano, Varco Sabino, Marcetelli, Pescorocchiano and Borgorose). In the coordinate system WGS 83 UTM 33N, the study area approximately extends from 4,692,537 m to 4,665,346 m north and from 330,012 m to 366,232 m east (Figure 1).

The climate is typical of central Italian Apennine mountain and hilly areas, with significant variations along a gradient; from sub-Mediterranean at low elevation (up to 800 m) to subalpine (>1800 m), through the sub-mountain (800–1200 m) and mountain (1200–1800 m) elevations. Annual precipitation ranged between 1161 and 1614 mm, mean annual temperature from 5.5 to 12.4 °C, and frequent frosts occur at higher altitudes in late spring [18].

The average elevation of the study area is about 1000 m a.s.l., ranging between 420 and 2225 m a.s.l.; western and southwestern aspects are prevalent (60% of the total surface), and slopes are 35% on average.

Forests cover most of the study area (61%; see Figure 1). At an altitude typically lying in the *Castanetum* phytoclimatic band, chestnut (*Castanea sativa*) and oaks (*Quercus cerris* and *Quercus pubescens)* dominate mixed coppice forests (Table 1). The shrub layer is located mainly in correspondence with abandoned fields and crops. Over 1000 m a.s.l., there is a gradual transition from mixed deciduous formations with sporadic beeches (*Fagus sylvatica*) to beech woods. In the study area, all broadleaved forests, including beech forests, are managed as coppice woods, resulting in complex and dense structures.

Pastures and open grasslands are sporadic and fragmented at altitude ranging between 1000 and 1200 m a.s.l., becoming homogeneous above the tree line. At the time of our survey, snow covered most of the study area above 1300 m a.s.l.

Cultivated areas (arable lands and permanent crops) are located in valleys near villages.

Seven main human settlements are included in the study area at lower altitude, with a very low human density of about 19.7 inhabitants km^2^. Scattered buildings are widespread, but not occupied all year long.

The main roads extend for 264 km, with a density 10% lower than the rest of the Rieti province, and are scarcely used.

Wild boar (*Sus scrofa*) and roe deer (*Capreolus capreolus*) are widespread in the study area, while red deer (*Cervus elaphus*) are still occasional but growing.

Extensive livestock farming is widespread within the study area; however, in winter, the presence of domestic ungulates is negligible. Only a few horses are free ranged in high pastures during the cold season without direct control.

**Figure 2:**
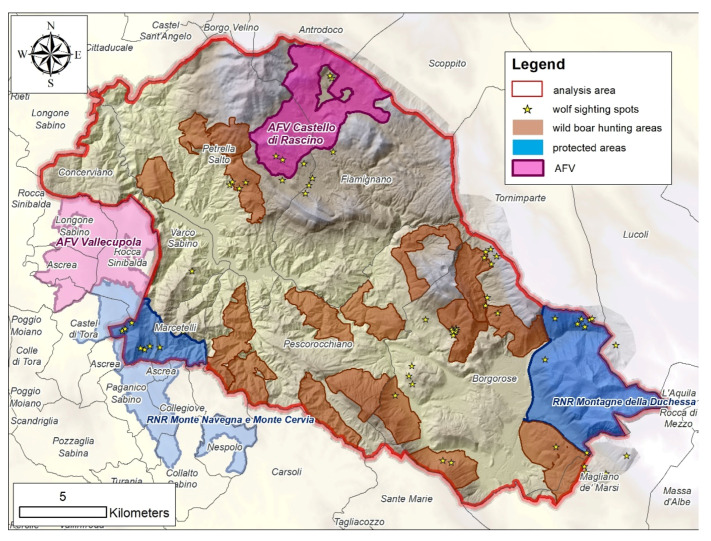
Maps of the territorial wildlife management plan. Brown polygons represent wild boar drive hunting areas. Blue polygons are the regional protected areas intersecting the study area. The private hunting areas (AFV) are identified in violet. Stars represent winter response sites (WRS).

A private hunting area (AFV Castello di Rascino, 28.56 km^2^) is placed in the northern part of the study area. Two regional parks (RNR Monte Navegna e Monte Cervia, RNR Montagne della Duchessa) intersect the study area, for a total surface of 46.6 km^2^ (9.3% of the study area) (Figure 2).

The study area is suitable for wolves, and their historical presence has been documented. A previous wolf-howling survey, involving a part of our study area, was performed in 2006–2007 [19], The same authors also reported the widespread presence of free ranging dogs.

### 2.2. Wolf Monitoring and Sampling Procedure

The wolf-howling survey was carried out in 2010 on 17 consecutive days of December. The method described by Apollonio et al. [20] was adapted, according to the protocol described by Gazzola et al. [3].

To elicit the howls of free ranging wolves, we emitted a recorded choral stimulus through a howling system composed of an MP3 reader, an SA15 amplifier, and a 15 W TC-15P loudspeaker early in the night (8.00–11.00 p.m.) [3]. The maximum sound level of the wolf vocalisations measured before the experiments at a distance of 2 m from the loudspeaker was 91.9 dBA.

With the help of the visibility tool of ArcGis 9.x^®^ (ESRI, Redlands, CA, USA), adjusted for sound wave diffraction, a total of 230 emission sites were chosen, in correspondence with acoustic vantage points, with the aim to maximize audibility and the extension of the overall covered area.

Standard operating procedures in ground-based radio tracking were adopted, aiming to minimize triangulation errors [21].

To maximize audibility and to allow for response site estimation, the survey was performed by an emitting and a listening group, distant about 1 km each other.

To limit the risk of pseudoreplication, we planned our field survey considering the results of our preliminary audibility analysis, thus ensuring the presence of acoustic barriers between consecutive emission sites. In the case of absence of effective barriers, successive emission stations were 5 km apart [13].

For both wolf and dog responses, the approximate direction and distance between the response source and listeners were recorded. The response locations were determined by triangulation and reported on appropriate layer maps, with the help of ArcGis 9.x^®^ (ESRI). Dog responses from uninhabited areas were attributed to free-ranging dogs.

With the aim to control for a reasonable triangulation error [20], we planned the successive assessment of response location characteristics within circular plots with 500 m radius (area of 78.5 hectares), centred on the triangulated point (response sample).

**Figure 3:**
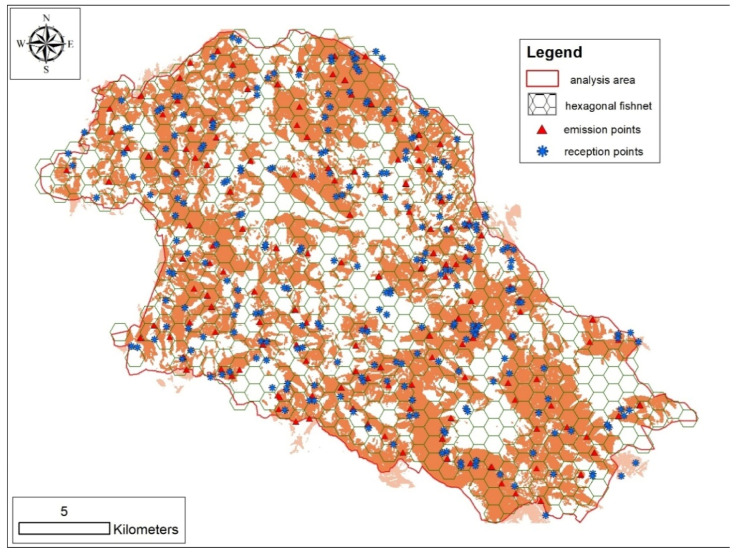
Map of the 0.78 km^2^ hexagonal meshes selected as availability sample, showing the distribution of emission (red triangles) and listening (blue asterisk) sites. Light brown polygons represent the resulting effective sampled areas based on audibility analysis.

Aiming to formulate a model of winter response sites with an approach presence (0.78 km^2^ circular plots around response locations) versus availability, a frame of 0.78 km^2^ hexagonal meshes [22] overlapping the entire study area was generated (Figure 3).

Meshes intersecting the study area for less than half of their surface were excluded. Of the remaining ones, only those overlapping more than the half the layer of the effective covered area were used to compose the availability sample.

At the scope to estimate the effective covered surface, considering the topography and the dense forest cover characterizing the study area, we assumed 3 km as the threshold of wolf listening ability [3] and 2 km as the threshold of howl audibility by humans [4,23]. Then, we considered the effect of physical barriers in limiting sound dispersion and audibility using the visibility tool of ArcGis, adjusted for sound wave diffraction (which is longer than the light one).

The estimated effective covered area was 267 km^2^ (53.4% of the entire study area; Figure 3), allowing us to evaluate the availability of sampled characteristics on 323 hexagons.

### 2.3. Antropogenic and Enviromental Predictors

Among all the environmental, landscape, and human-related factors affecting wolf presence and responsiveness, those potentially related to both prey availability and hunting efficiency may be relevant during late fall and winter [1]. In this view, land-cover types and use have been reported as a crucial driver [24,25,26,27,28,29,30], while topography [30,31], natural and anthropogenic landscape features [32,33,34,35,36], human disturbance, land-use planning, and wildlife management [27,31,37,38,39,40,41] also play important roles.

Accordingly, we entered eight land cover types, four topographic factors, four human disturbance factors, four territorial planning and wildlife management factors, and three landscape features (Table 2) as possible predictors of WRS.

### 2.4. Statistical Analysis

All statistical analyses were performed using the R statistical software [44].

We formulated the model of wolf howling responsiveness in winter following an approach considering presence (WRS) versus availability.

Hotelling’s two-sample T^2^ statistic (T^2^) [45] was first used to test the differences between the multivariate means of the two groups, where the first and the second group were composed, respectively, of circular plots surrounding the estimated response locations (WRS) and the hexagons overlapping the effective sampled area selected as representative of the overall available conditions (availability sample).

The T^2^ statistic rejects the hypothesis that the means of the two groups are equal for large values of the statistic reported below [46]:

(*n*_1_*n*_2_/*n*)(**m**_1_ − **m**_2_)^′^**S**^−1^(**m**_1_ − **m**_2_), (1)

where *n*_1_, *n*_2_ and **m**_1_, **m**_2_ are the sizes and the sample means of the two groups, *n* is the number of observations, and **S** is the pooled sample covariance matrix.

The T^2^ statistic was computed with the R function hotelling.test {Hotelling} [47], by considering all variables. We also conducted the same test on different categories of the variables.

Hotelling’s test does not give information about the role of each factor in determining the difference between two samples. Then, a logistic LASSO regression [48] was performed.

In a linear model containing many predictors, standard OLS (ordinary least squares) parameter estimates have large variances, thus making the model unreliable. In order to address this problem, we used the LASSO regression, a technique which decreases such variance at the cost of introducing some bias. A good bias–variance trade-off minimizes the model’s total error.

LASSO is a shrinkage method, and its estimate is defined as

(2) $$\hat{\beta }{\;}^{lasso\;}=argmin_{\beta }\overset{N}{\underset{i=1}{\sum }}{\left(y_{i}-\beta_{0}-\overset{p}{\underset{j=1}{\sum }}x_{ij}\beta_{j}\right)}^{2},\;$$

where

(3) $$\overset{p}{\underset{j=1}{\sum }}\left|\beta_{j}\right|\leq t.$$

The Lagrangian form of the LASSO problem is as follows:

(4) $$\hat{\beta }{\;}^{lasso\;}=argmin_{\beta }\left\{\frac{1}{2}\overset{n}{\underset{i=1}{\sum }}{\left(y_{i}-\beta_{0}-\overset{p}{\underset{j=1}{\sum }}x_{ij}\beta_{j}\right)}^{2}+\lambda \overset{p}{\underset{j=1}{\sum }}\left|\beta_{j}\right|\right\}.$$

In the form above, the sum of the absolute values of the coefficients is the LASSO penalty (L1) [48]:

(5) $$L1=\overset{p}{\underset{j=1}{\sum }}\left|\beta_{j}\right|.$$

In this way, the solution is non-linear in $y_{i}$, resulting in a quadratic programming problem with no closed-form expression.

The tuning parameter, lambda, measures the amount of shrinkage. It is chosen by cross-validation and controls the strength of the L1 regularization penalty. When lambda equals zero, no parameter is eliminated. As lambda increases, more and more coefficients are set to zero, while the bias increases. When lambda tends to infinity, all coefficients are eliminated. On the other hand, when lambda decreases, the variance increases.

Thus, LASSO is a combination of both shrinkage and selection of variables.

We used the R function glmnet {glmnet} [49] with a logistic regression model, as the dependent variable was dichotomous. This function fits a generalized linear model using a penalized maximum likelihood.

Before performing LASSO regression, we selected the lambda value by means of cross-validation, which is an estimate of the expected generalization error for each lambda, and is usually chosen to be the minimizer of this estimate by applying the cv.glmnet {glmnet} R function.

This function returns two values of lambda: (i) lambda.min, which minimizes the binomial deviance, and (ii) lambda.1se, which is a heuristic choice of lambda producing a less complex model, for which the performance (in terms of estimated expected generalization error) is within one standard error of the minimum. As the folds (i.e., sections in which the data are divided, ensuring that each fold is used as a testing set at some point) are selected at random, the cv.glmnet {glmnet} outputs are also random. We reduced this randomness by running the function cv.glmnet many times, carrying out 100 cross-validations and averaging the error curves.

## 3. Results

Over the 230 howling sessions performed, 58 wolf replies were recorded (response rate = 25.21%).

To provide a synthetic description of WRS characteristics versus the available ones, the descriptive statistics of the investigated continuous variables are reported in the table below (Table 3).

WRS were located at higher altitudes, in places where the slope and aspect favour irradiation and higher heat load indices (HLI). Furthermore, WRS were characterized by lower cover level (broad-leaved forest + scrublands) than availability, except for coniferous forests which, to the contrary, were more represented in the former case. The land-use types subjected to intensive anthropic use (cultivated lands and fruit chestnut) were less represented in WRS, with respect to availability, as well as main roads, urban areas, and water bodies that, in the present case, were located close to the latter.

Free ranging dog replies (response rate = 49.13%), belonging to the human disturbance factor category (Table 2), involved 29.31% of the 58 WRS and the 65.02% of the 323 hexagons matching the available conditions.

Regarding the time since the last hunting action, included in the TPWM factor category (Table 2), we found wolf replies to be more frequent (58% of WRS) at sites where the last wild boar drive hunting actions occurred at least 28 h before the survey time.

### 3.1. Groups Comparisons (WRS vs. Availability)—Multivariate Test

Consistent with the suggestion of the descriptive statistics (Table 4), the multivariate T^2^ test showed significant differences (*p* < 0.001) between the mean vectors (all variables) of the two compared groups (WRS vs. availability).

The same test performed separately over each predictor category confirmed the existence of diverging conditions between WRS and availability (Table 4) at scales of analysis.

### 3.2. Model Development—Logistic LASSO Regression

**Figure 4:**
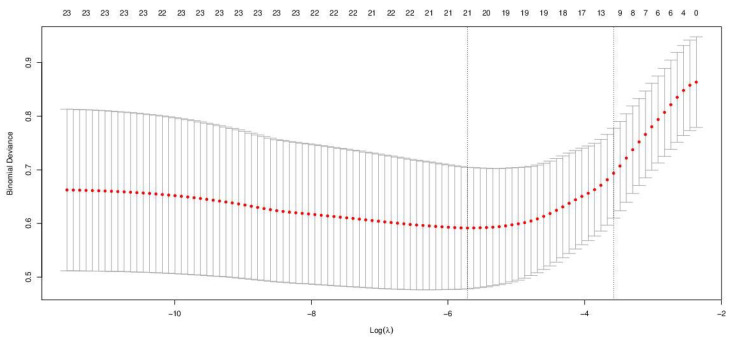
Graphical representation of the cross-validation outputs. The lambda interval delimited by the two vertical lines identifies the optimal selection range.

The outputs of the cross-validation, aimed to select the best lambda value for model development, are reported in Table 5 and graphically represented in Figure 4.

On the basis of the outputs above, we selected the lambda value of 0.0025 (lamba.sel) to perform LASSO (Table 6), being the smallest of the hundred lambda values generated by cross-validation.

The logistic model explained 50.22% of the deviance of the response variables and, with an R^2^ value of 0.53, it showed a good fitting ability.

The LASSO model developed with the selected lambda retained 21 non-null parameters, excluding only principal and secondary road density among human disturbance factors. The LASSO-estimated non-null parameters and the respective odds ratios are reported in Table 7.

Among land-cover factors, only conifers positively (odds = 1.028) affected the occurrence of WRS, while other wood cover types (broadleaved-forests and scrublands), as well as open areas (pastures and natural grasslands) and bare ground, had negative effects.

All the variables strictly associated to human presence and activities, such as urban areas (odds = 0.873), cultivated lands (odds = 0.946), and chestnut cultivation (odds = 0.846), reduced the likelihood of obtaining a wolf response.

Among human disturbance factors, the role of free-ranging dog reply was crucial (odds = 0.207) in determining a strong reduction (−79.25%) of wolf probability of responses.

Protected area occurrence positively affected (odds = 1.014) the WRS, as well as the presence of wild boar drive-hunting areas (odds = 1.015) and the time since the last hunt (odds = 1.019).

Both average altitude (odds = 1.001) and slope (odds = 1.014), commonly inversely related with the intensity of human activities, positively affected the occurrence of WRS. Both overall and within the topographic factor group, the most explanatory variable was the heat load index (odds = 114.4859), which determined an impressive increase (+11,348.54%) in the probability of WRS occurrence.

Landscape features also affected wolf responsiveness in winter, with positive effects due to edge density (odds = 1.828) and patch richness (odds = 1.153), and a negative contribution of the total patch number (odds = 0.968).

## 4. Discussion

During our late fall/winter survey, wolves howled in response to simulated vocal stimuli in particular spots (winter response sites, WRS) characterized by environmental and human-related conditions, which significantly diverged with respect to the available ones (Table 4), suggesting that WRS are non-randomly located within the overall suitable territory, but were selected by wolves for some reason. In particular, our results highlighted the positive role of several factors potentially affecting food (carrion and prey) availability and prey catchability by wolves (Table 7).

In agreement with our results, Harrington and Mech [1] highlighted that, during the non-rendezvous period (particularly in December–February), wolves commonly limit their reaction to vocal stimuli when kills or carrions are present at or near the response sites. Furthermore, several studies [15,16,17,50] have verified that the place where a kill occurs is affected by habitat features representing the best trade-off between prey availability and the probability of a final successful kill.

Land-cover has been commonly reported to be an important factor category affecting the abundance of wild ungulates—and, consequently, the presence of wolves—as a result of different predator–prey encounter probabilities [14,15]. Forest cover, in particular, was reported to be a crucial driver of wolf presence, being directly associated with trophic resource availability [8]; however, the latter is only a component of the predation hierarchical process described by McPhee et al. [14]. Indeed, where prey are available, the success of a hunt strongly depends, at the local scale, on habitat and landscape features that contextually favour predation strategies and hinder the escape of the prey [15,16,17,50,51,52].

In our study area, coppice broadleaved forests and scrublands are widely distributed under the tree line (Table 1), potentially shaping homogeneous wild prey availability. This negative effect on WRS occurrence could more likely be attributed to local conditions that limit the occasion of a successful kill than to lower predator–prey encounter probability.

In agreement with our results, previous works [50,53] have reported that, in dense forest cover, wild ungulates are more wary than in open areas and that this condition favours predator detection due to their noisier approach [54]; moreover, previous studies have shown that roe deer presence in the wolf diet increased with an increase in open areas [55].

During winter, evergreen forests (i.e., conifers)—which, in our case, positively affected WRS occurrence—play the role of winter refuge, determining local thermal and trophic favourable conditions for prey species [56,57,58,59]. Coniferous forest, normally associated with steep slopes, are also considered elective winter escape terrain for wild ungulates to avoid human disturbance [59,60], determining a high encounter rate with prey species. Furthermore, scarce undergrowth and the relatively open structure of pinewoods in the study area can favour prey detectability, approach, and pursuit. The common presence of fallen trees in mountain coniferous forest can also provide useful obstacles against prey escape, favouring the final attack [17].

Several studies have reported positive relationships between open habitats and wolf presence [61], kills, and scavenging sites [50,52,62,63], suggesting that extended open areas favour coursing behaviour, allowing wolves to engage in cooperative hunting strategies and to better choose vulnerable individuals [52,63]. Differently, in our case study, open habitats (i.e., pastures and natural grasslands) negatively affected wolf responsiveness in winter. This evidence should be analysed, taking into account the “optimal foraging theory” [64] and, consequently, the seasonality of high quality forage. Although open habitats expose grazers to high predation risk, determining a higher encounter rate with predators as they are more easily detectable than in covered habitats, when high quality forage is available, the risk is justified by a positive cost–benefit balance [65]. However, in this case study, a howling survey was performed in winter, when snow cover is extensive and grazers commonly prefer to stay inside or along the edges of thermal refuges as evergreen hiding-cover [56,57,58,59]. Furthermore, as recorded in a similar central Apennine area [66], wolves avoided open scavenging sites where free-ranging dogs are present.

Bare-ground and sparsely vegetated areas present the limits already discussed for open areas. In addition, these typologies are typically limited to steep slopes, where the superficial rock layer determines scarce herbaceous cover of poor quality and, thus, low attractiveness for grazers.

Cultivated areas were negatively related to WRS. Indeed, in mountainous areas, agriculture is relict to valley areas close to human settlements, which are generally less used by wolves than areas characterized by a low anthropization index [7,27,31]. Chestnut (*Castanea sativa*) orchards are also intensively managed for fruit production. Then, undergrowth preparation and harvesting operations, normally involving 40–60 days from mid-September to the end of October, could determine unfavourable local conditions for both prey and predators.

Although previous works have highlighted the positive effects of waterbodies and watercourses on both prey abundance [24] and kill success [14,17], as they are valuable resources during the dry seasons and serve as physical obstacles to prevent prey escaping, in our case study, waterbodies had a negative contribution to the prediction of WRS. A possible explanation is that our survey was performed during late fall, when water is normally a widespread available resource within the study area, potentially shaping a homogeneous distribution of temporary watering points. Wolf presence has also been reported to be affected by waterbodies only during the dry season, corresponding to denning [25,27]. Furthermore, in our study area, the only waterbody large enough to represent a physical obstacle to prey escape is the Lake of Salto, located at a lower altitude in a densely forested area characterized by a higher level of anthropization than the average of the study area. These conditions can determine, at the local scale, lower opportunity from the predator’s point of view [50].

As discussed above, some of the land-cover factors normally reported to affect both prey availability and detectability also affected the location of wolf response in winter (WRS). However, the probability of a final successful kill is mediated, at the local scale, by the topography [30,31,50,67], natural and anthropogenic landscape features [32,33,34,35,36], human-related factors, territorial planning, and wildlife management [27,31,37,38,39,40,41]. Some of these also determine favourable conditions, in terms of carrion availability [33,62,66,68].

In agreement with previous works describing both prey abundance and catchability as being positively affected by habitat edges [17,69], we found patch richness and edge density to be positively associated with WRS occurrence.

Among topographic factors, both altitude and slope were retained by Lasso as favourable predictors of WRS (Table 5).

Previous studies have indicated altitude as a crucial driver of wolf occurrence, with presences increasing with altitude, explaining that wolves tend to avoid areas at low altitudes, where human settlements and activities normally take place [7,27,31].

In the other hand, during winter, prey availability can decrease at high altitudes as wild ungulates perform altitudinal migration to low-elevation areas, according to the severity of winter and snow depth [70,71]. However, Ramanzin et al. [72] argued that seasonal movements occur almost exclusively in years of heavy snowfalls. In addition, Cagnacci et al. [67] concluded that these movements strongly depend on the interaction between snow depth and slope.

Our results suggested that slope contributes more than altitude in determining the occurrence of WRS. In agreements with our observation, Torretta et al. [50] recorded, in a mountainous area in northern Italy, the positive effect of steep surfaces on kill site locations, regardless of altitude. According to the authors, steep slopes offer a vantage point from which wolves can scan prey groups and identify the most vulnerable individuals. Furthermore, gullies, ravines, and rock walls, which are frequent in steep mountainous areas, represent typical terrain traps where prey are forced to slow down, offering good opportunities to attempt a successful final attack [73].

Among all the considered factors, the heat load index (HLI) was the most explanatory (odds = 114.485) with respect to the occurrence of WRS, reinforcing the importance of thermal refuges in winter, as already discussed above in connection with the positive role of conifers. In general, all the animals chose resting sites and adopted circadian rhythms searching for favourable temperature regimes [74]. In winter and, in particular, in mountainous regions, prey species (e.g., roe deer) concentrate their activities at dawn and during the daylight while, in the night, when wolves are more active [50], they prefer rest in favourable thermal refuges to reduce energy cost and heat loss.

With respect to the contribution of human disturbance factors, previous studies have concluded that wolves prefer to avoid areas with high road density and human settlements [50,75]. Accordingly, in our study area, we recorded the negative effect of urban areas, but no predictive importance was attributed to these factors. In agreement with our results, several works have confirmed that the perception of human disturbance depends on the degree of utilization [33]. Wolves respond to human activities by adopting a complementary spatiotemporal pattern; in particular, during the night-time, when human presence is low, they can even take advantage by low traffic roads [15,33,36].

Among human disturbance factors, particularly interesting was the effect of free-ranging dogs on WRS. Indeed, dog replies had a strong negative effect (odds = 0.207) on the presence of responding wolves. When dogs reacted to our simulated howls, wolves rarely responded (7.5%). Regarding this interaction, Molnar et al. [76] reported that, in the nearby Abruzzo, Lazio, and Molise National Park (central Apennine, Italy), where a widespread presence of free-ranging dogs was ascertained, interspecific competition determined an impressive increase in stress levels in wolves. Coherently, Mancinelli [66] found, in the same area of sympatry, that wolves avoided open kills or scavenging sites due to frequent use by free-ranging dogs, suggesting spatial segregation between the two species. Another recent study [77] reported a strong negative effect of both feral and owned free-ranging dogs on the occupancy of wild carnivores; however, in the present case study, we could not determine whether the wolves did not howl due to absence, as a consequence of spatial segregation from dogs, or because they preferred to stay silent, aiming to keep a resource hidden from non-conspecific competitors.

Drive hunting is commonly considered a wildlife disturbance factor [41]. Indeed, according to the “ecology of fear” [64], hunting shifts wild ungulate home ranges towards hunting ban areas, influencing their abundance at the local scale [37,38,39]. On the other hand, hunting can also provide easy accessible food supply [25,68,78], providing injured animals and carrion for carnivores [25,78]. According to the knowledge above, we found that the occurrence of WRS was positively affected by both protected and drive hunting areas. Evidently, on one hand, the refuge effect generates a shift of prey distribution and abundance, determining high predator–prey encounter probability inside or near protected areas; on the other hand, wild boar drive hunting areas provides easily accessible low energy cost food. Furthermore, the regular utilization of the hunting zone (three times per week) from November to January favours the adoption of complementary spatiotemporal patterns. Indeed, we found a positive effect of the time since the last hunt, suggesting that wolves avoided approaching hunting areas in search of carrion on the day the hunt was carried out.

In conclusion, we demonstrated that WRS are characterized by several landscape features, anthropogenic and environmental factors often related to food (prey and carrion) availability and hunting efficiency. However, in wider and different areas, wolves equipped with VHF or GPS tracking systems can allow for checking, directly in the field, the real nature of the resources motivating their reply in late fall and winter.

## Figures and Tables

**Table 1:** Land-covers of the study area.

Name	Description	Area (km^2^)	%
Urban areas	Human settlements	6.94	1.39
Principal roads	Main paved roads	6.29	1.26
Secondary roads	Gravel roads	2.44	0.49
Cultivated lands	Arable lands and permanent crops	50.16	10.02
Open areas	Pastures and natural grassland	74.58	14.90
Broad-leaved forests	Oak, chestnut, beech, and other mixed coppice woods	299.61	59.87
Coniferous forest	Black pine	6.72	1.34
Scrubland	Bushes and shrubs	39.56	7.91
Bare grounds	Rocks and sparsely vegetated areas	2.49	0.50
Water bodies	Lake and rivers	7.45	1.49
Fruit chestnuts	Cultivated woods for fruits production	4.16	0.83
Total		500.40	100.00

**Table 2:** List of the tested predictors.

Predictor Categories	Name	Description	Unit
Land cover (LC)	Cultivated lands	Arable lands and permanent crops	%
	Open areas	Pastures and natural grassland	%
	Broad-leaved forests		%
	Coniferous forest		%
	Scrubland	Bushes and shrubs	%
	Bare grounds	Rocks and sparsely vegetated areas	%
	Water bodies	Lake and rivers	%
	Fruit chestnuts		%
Topography (TPG)	Average slope		%
	HLI index	Heat load index—aspect rescaling equation ^1^	
	Average altitude		m a.s.l.
	Roughness	Index of topographic heterogeneity ^2^	
Human disturbance (HD)	Urban areas Main roads	Villages, transport, industrial/commercial	%
		Density of paved roads	km km^-2^
	Secondary roads	Density of gravel roads	km km^-2^
	Dogs	Dogs responding to simulated howls	yes/not
Territorial planning and wildlife management (TPWM)	Protected areas	Hunting ban regional protected areas	%
	Drive hunting areas	Specifically assigned for wild boar drive hunting	%
	Private hunting areas	Privately managed for hunting purpose	%
	Time since the last hunt	Time since the last hunt occurred within wild boar drive hunting areas	hours ^3^
Landscape features (LF)	Patches number	Number of fragmented patches	n°
	Patch richness Edge density	Number of land cover types	n°
		Ecotone between closed ^4^ and open habitats	km km^−2^

^1^ McCune and Keon (2002) [42] formulated an equation for potential annual direct incident radiation and heat load index (HLI), which rescales the aspect such that the highest values (1) are southwest, and the lowest values (0) are northeast. This method accounts also for: the steepness of slopes; ^2^ roughness expresses the amount of elevation difference between adjacent cells of a DEM [43]; ^3^ three time intervals: 4, 28, and 52 h; ^4^ closed areas = sum of forests and scrublands.

**Table 3:** Characteristics (mean ± SD) of the investigated variables for WRS (*n* = 58) and availability (*n* = 323).

Predictor Categories	Variables	WRS		Availability	
		Mean	SD	Mean	SD
LC	Cultivated lands	8.81	15.14	12.65	19.34
	Open areas	8.49	13.22	8.42	10.56
	Broad-leaved forests	38.12	28.66	56.64	27.48
	Coniferous forest	2.32	6.49	0.44	2.18
	Scrubland	8.29	10.63	8.23	9.72
	Bare grounds	6.11	12.56	6.68	13.80
	Water bodies	0.09	0.49	2.25	11.48
	Fruit chestnuts	0.00	0.00	1.08	5.30
TPG	Average slope	39.18	13.22	34.16	13.52
	HLI index	0.79	0.04	0.76	0.04
	Average altitude	1175.64	226.81	973.02	296.01
	Roughness	69,487.98	48,141.72	58,968.91	45,288.13
HD	Urban areas Principal roads	0.48	1.35	1.70	4.30
		0.72	1.54	1.61	2.07
	Secondary roads	0.91	0.91	0.85	0.99
TPWM	Protected areas	27.44	41.80	7.77	25.00
	Drive hunting areas	17.22	30.15	17.56	31.87
	Private hunting areas	4.70	18.83	7.77	24.88
LF	Patches number	24.97	11.73	31.14	16.12
	Patch richness Edge density	6.90	1.85	7.15	2.24
		5.93	3.53	6.35	4.07

**Table 4:** Hotelling T^2^ statistic. Significant differences are listed in bold.

Predictor Category	Statistic	*p*
All variables	12.428	0.000
LC	19.580	0.000
TPG	6.447	0.000
HD	7.506	0.000
TPWM	6.504	0.000
LF	3.365	0.005

**Table 5:** Cross-validation outputs.

	Lambda	Binomial Deviance	Standard Error	No. of Non-Null Parameters
Lambda.min	0.0033	0.5915	0.1133	21
Lambda.1se	0.0280	0.6936	0.0838	12

**Table 6:** Values of the LASSO regression models developed with the selected lambda.

Lambda	%DEV	R^2^	No. of Non-Null Parameters
0.0025	0.5022	0.5291	21

**Table 7:** Logistic LASSO regression-estimated coefficients and their ODDS-based interpretation.

Predictor Category	Name	Coefficient	ODDS	Increase or Decrease in the ODDS ^1^
Intercept		1.274	3.577	
LC	Cultivated lands	−0.05532	0.946	−5.382%
	Open areas	−0.09742	0.907	−9.282%
	Broad-leaved forests	−0.09941	0.905	−9.462%
	Coniferous forest	0.02733	1.028	+2.771%
	Scrubland	−0.06320	0.939	−6.125%
	Bare grounds	−0.08875	0.915	−8.493%
	Water bodies	−0.09636	0.908	−9.187%
	Fruit chestnuts	−0.16660	0.846	−15.349%
TPG	Average slope	0.01412	1.014	+1.422%
	HLI index	4.74000	114.485	+11,348.540%
	Average altitude	0.08186	1.001	+0.082%
	Roughness	−0.08278	0.999	−0.000%
HD	Urban areas Principal roads	−0.13550	0.873	−12.674%
		-	-	-
	Secondary roads	-	-	-
	Dogs	−1.57700	0.207	−79.250%
TPWM	Protected areas	0.01367	1.014	+1.376%
	Drive hunting areas	0.01511	1.015	+1.522%
	Private hunting areas	−0.01376	0.986	−1.367%
	Time since the last hunt	0.01883	1.019	+1.901%
LF	Patches number	−0.03198	0.968	−3.147%
	Patch richness Edge density	0.14270	1.153	+15.339%
		0.01819	1.018	+1.828%

^1^ ±absolute value (1-ODDS RATIO) * 100 = effect of each factor unit increase on the probability (%) of obtaining a positive response by wolves.
